# A Demonstration of the Curability of Pulmonary Consumption in All Its Stages

**Published:** 1844-09

**Authors:** 


### REVIEWS.

Art. III.—*A Demonstration of the Curability of Pulmonary Consumption in all its Stages. Comprising an Inquiry into the Nature, Causes, Symptoms, Treatment, and Prevention of Tuberculous Diseases in general.* By William A. M'Dowell, M.D. Louisville, Ky. Prentice & Weissinger. 1843. pp. 269. 8vo.

We believe there is but one opinion among the professional readers of this work as to its merits. So far as it has gained the attention of medical men, the sentiment almost universally felt towards it, if we are not greatly mistaken, is one of no very high respect. The author has complained, indeed, that his work has been neglected by the profession, and one can readily suppose that the complaint is not without good grounds. For ourselves, we confess that we have felt a decided repugnance to reviewing it. We have felt most averse to disturbing the profound repose which we imagined it was enjoying, and was likely to continue to enjoy, on the shelves of the book-sellers. Cherishing no other feeling towards the author than one of good will, not only were we disposed to let his book rest, but we were even amiable enough to admit into our journal, nearly a year ago, a communication from him in relation to it, and to the merits of the method of cure therein set forth, which must have appeared to our readers much in the light of a puff direct. We have reason to believe that that communication has been productive of mischief. From its phraseology, readers have been led to suppose that the editors of this journal had in their possession decisive testimony to the efficacy of Dr. M'Dowell's mode of treating consumption. The '*demonstration* of the curability of consumption *in all its stages*,' sustained as it was by what seemed to be our silent assent, has brought persons far gone in that disease to this city, only to die in a few weeks among strangers. We acquit Dr. M'Dowell of all purpose to deceive. In affixing such a title-page to his book, empirical as it strikes one, we can believe that he was stating the honest convictions of his understanding. Now and then, at long intervals, it is the good fortune of the practitioner to see a case of pulmonary consumtion cured, in what pathologists term the last stage of the disease. In asserting then, that the complaint was curable 'in all its stages,' Dr. M. set forth nothing more than the concurrent opinion of of the profession. We will allow that this was all that he meant to proclaim.

Such, however, has not been the impression of the public, concerning his pretensions and promises. He has been understood 'in the momentous demonstration which he claims to have made,' to teach that he cures consumption in its last and worst stages—that his 'course of treatment', in a word, 'is as specific for the control of tubercular diseases, as the use of quinine is in febrile diseases.' This has gone abroad in the pages of this journal, and not a note of warning or disapprobation, up to this time, has followed, to put our readers on their guard. We feel that an apology is due them—not so much for a failure to notice the work, as for admitting the advertisement to which reference has been made. So we propose now making amends for both delinquencies, as far as lies in our power, by exhibiting the book and its pretensions to a 'momentous demonstration,' in their true colors. Dr. M'D. has challenged investigation, and cannot complain if his claims to discovery are severely scrutinized. Moreover, he has had the benefit, for nearly a twelvemonth, of all the currency that our journal could give to his claims, and cannot therefore object, if we come forward thus late in the day and declare what is our estimate of the validity of those claims.

The pretensions of the work, as the reader has perceived by the title-page, are high. It professes to demonstrate, that consumption is curable in all its stages. That is a good deal. But it farther intimates, that the author was the first to make this momentous discovery. Nor is this all. It is by a novel and happy combination of remedies made by himself, that a control over tubercular diseases, perfect as that of quinine over intermittent fever, has been gained. In so many words; Dr. M'Dowell professes to have discovered a specific for consumption. Well may he wonder that the announcement of such a discovery has attracted so little attention. Only one explanation of it can be given—his demonstration has not been satisfactory. The profession has not given credit to the discovery. So many "cures for consumption" are before the public, that any new one works its way slowly to general notoriety. But, without further preface, let us proceed to notice Dr. M'Dowell's claims to originality.

The author shall speak for himself. His claims are set forth in the following extracts:

"My mode of treatment may be liable to exceptions, because it is somewhat novel and peculiar.

"The claim which I submit to the profession for a favorable consideration of treatment, is based on the fact that the result of my investigations and experience enables me to define a course of treatment which I feel confident will be found as specific for the control of tubercular diseases, as the use of quinine is in febrile diseases.

"These, (the experience and observation of others) together with the well-known character and modus operandi of the medicines I recommend, will, I trust, without attaching undue weight to my own experience, clearly demonstrate that consumption is a disease as curable as any other to be found in the therapeutical catalogue."

We notice first, the claim to originality in regarding consumption as curable in all its stages; and we regret to say that the claim is one which the profession will not be able to allow. This idea did not originate with our author, and is far from being peculiar to him. It is not a little singular that he should ever have fancied that it was. As to the last and worst stage of the disease, he indeed admits, that the ablest pathologists teach that it is curable. If he had consulted a very popular and accessible work, Stokes and Bell's Lectures (vol. 2, pp. 363, 365, 368) he would have found high authority for the curableness of consumption in its earlier stages. Other authorities might be mentioned, but we will not stop to quote them. These are sufficient for our purpose, since they make it clear that the doctrine taught in the work before us, amounting, as the author supposes, to a demonstration, is not new in the profession. But we pass from this claim, which, after all, is of but secondary importance, to the higher one of novelty and peculiar success of treatment. We have seen that Dr. M'Dowell ascribes to his method of cure even *specific* virtues. Let us see, if we can, in what the peculiarity of his system consists. What is it?

Really, we are at a loss for an answer. We will give the one which our author himself furnishes. He says:

"My *only peculiarity* consists in the application of therapeutical agents, more than usually in accordance with pathological indications that have been ascertained from actual autoptical observation of the fluids as well as the solids of the system."

The meaning of this is, that Dr. M'Dowell treats consumption more scientifically than anybody else. That is his peculiarity. He employs no new remedies, but applies the old ones more in accordance with pathological indications. *Iron* and *common salt* constitute his sheet-anchor. If they do not cure, we understand him to admit that the cure cannot be accomplished at all. But to do good, it would appear that their action upon the system must be understood. He remarks, on this subject—

"The especial *modus operandi* of the principal articles I use in the cure of consumption, more especially the powerful anti-tubercular efficacy of common salt, has heretofore, as I am aware, been but little, if at all, adverted to."

The author seems to be aware of the fact, that "brandy and salt" have been making a prodigious noise in the country, for years past, as the "sovereignest balm" yet discovered for all the ills that assail poor humanity; but, then, their *modus operandi* was not adverted to or understood. Hence their failure.

To us, there is something almost ridiculous in this. After such a flourish of trumpets in the introduction, about novelty and peculiarity of practice, the only novelty brought to light in the sequel, consists in a more scientific use of iron and salt—a more correct appreciation of their *modus operandi.* And we are asked to believe that these articles, thus guarded, are as specific against consumption, as quinine is against fever and ague! Nor would a knowledge of their *modus operandi* seem to be indispensable to their success; for after all that the author had previously urged upon that point, he winds up his remarks on iron with these words:

"But whether this be the *modus operandi* or not, we have at least abundant matter-of-fact evidence that iron is productive of each of these important changes in the system, compared with which all speculation upon *modus operandi* is *idle* and *profitless.*"

If the reader should now come back upon us with the question, *where, then, is the peculiarity?* we should be obliged to answer, *we do not know.* It is not the iron and common salt, for they are old and common. And it would be trifling to say that it is to be found in the author's speculation upon their *modus operandi*, for that he has pronounced "idle and profitless."

We respectfully submit whether there is anything in this method of cure to flatter the hopes of the consumptive? We submit, especially, whether there is anything in it to tempt patients, with the disease far advanced, to forsake their family physicians, and all the comforts of home, and come to pass their last weeks among strangers in a tavern?

It does not comport with the end we had in view in writing this brief article, to consume time and space by dwelling on the pathology of our author, or the style in which his book is written. The latter, it must be admitted, is very much his own. We presume, no one will dispute with him the title to it. But he will find it difficult to persuade any one, that he has the shadow of a claim to originality in his views concerning the pathology of consumption. No one, especially, who reads his book with tolerable attention will give him credit for it. It will be impossible; for it must be perceived that the opinions laid down on one page as original and peculiar to the author, are referred to on subsequent ones as having been long held by others.

In this manner our author unceremoniously sets aside, in one paragraph of his book, claims which he had set up in another, and in the end makes it perfectly manifest, that he has no claims to originality either of pathology or therapeutics. No doubt, he treats consumption with as much success as other physicians; but he has certainly written, concerning it, one of the most bungling, blundering volumes that ever was called forth by the subject. He has, possibly, seen cases of consumption cured; but we are yet to learn what new agent or process he has added to our professional resources. His "demonstration" amounts to nothing. It does not even embrace the whole of the little evidence extant, that tubercular phthisis has been cured in some happy instances.

With this brief exposition, we dismiss the work. We do not suppose that any professional opinion will have much weight with the victims of consumption. They will still listen to the promises of cure, from whatever quarter they may come. Theirs is a hope which nothing but the grave can extinguish. But we have written this for professional readers; and if, hereafter, they recommend their patients with tuberculous lungs to visit this city, in search of the healing virtues of iron and table salt, they will do so with their eyes open.

